# Comparative lethality kinetic curves and predictive models of *F*-value for *Listeria monocytogenes* using different sanitizers

**DOI:** 10.1002/fsn3.5

**Published:** 2013-01-08

**Authors:** Cezar A Beltrame, Gabriela B Kubiak, Ieda Rottava, Geciane Toniazzo, Rogério L Cansian, Lindomar A Lerin, Débora de Oliveira, Helen Treichel

**Affiliations:** 1 Department of Food Engineering, URI – Campus de Erechim, Av. Sete de Setembro, 1621, Erechim, Rio Grande do Sul, 99700-000, Brazil; 2 Departamento de Engenharia Química e Engenharia de Alimentos, Universidade Federal de Santa Catarina, UFSC, Campus Universitá rioBairro Trindade, Caixa Postal 476, Florianó polis, Santa Catarina, 88040-900, Brazil; 3 Universidade Federal da Fronteira Sul – Campus de ErechimAv. Dom Joã o Hoffmann, 313, Erechim, Rio Grande do Sul, 99700-000, Brazil

**Keywords:** Chlorhexidine, lethality, *Listeria monocytogenes*, organic acid, peracetic acid

## Abstract

The objective of this work was to evaluate the kinetic of inactivation of *Listeria monocytogenes* using peracetic acid, chlorhexidine, and organic acids as active agent, determining the respective *D*-, *Z*-, and *F*-values. From our knowledge, these important results from an industrial view point are not available in the current literature, mainly for organic acids, pointing out the main contribution of the present work. Lower *D*-values were obtained for peracetic acid and chlorhexidine, compared with the organic acids. For the reduction of 6 log_10_ of *L. monocytogenes* using peracetic acid, at 0.2, 0.1, and 0.05% are necessary 7.08, 31.08, and 130.44 min of contact, respectively. The mathematical models of *F*-values showed that at concentrations lower than 0.15% one can verify an exponential increase in *F*-values, for both de chlorhexidine and peracetic acid. The organic acids presented a linear behavior, showing slight variation in *F*-values, is even more effective in under dosage. The results obtained are of fundamental importance in terms of industrial strategy for sanitization procedure, permitting to choose the best relation product concentration/exposure time, aiming at reducing costs without compromising the disinfectant efficiency.

## Practical Applications

The results obtained in the present work are of industrial importance and not available in the current literature, mainly for organic acids. The results obtained are of fundamental importance in terms of industrial strategy for sanitization procedure, permitting to choose the best relation product concentration/exposure time, aiming at reducing costs without compromising the disinfectant efficiency.

## Introduction

*Listeria monocytogenes* has been considered the most important pathogenic microorganism transmitted by food due to the high death rate in risk group (Thévenot et al. 2005), and its ability of surviving in adverse conditions (Varabioff 1992; Incze 1998; Bolton and Frank 1999; Bonnet and Montville 2005). Generally, these microorganisms are found in the natural ambient of food processing as a biofilm able of reproducing at refrigerator temperatures (Muriama 1996; Ibusquiza et al. 2011). They have been isolated from the soil, vegetation, domestic and industrial residues, water and food industries (McGlaughlin 1987; Kastbjerg and Gram 2009).

Peracetic acid and chlorhexidine have been widely used in food industries and evaluated for their effect under different microorganisms (Frank et al. 2003; González-Fandos et al. 2005; Pan et al. 2006; Aarnisalo et al. 2007). The organic acids have been used directly in food products in the control of *Salmonella* spp., while also presents effects under other bacteria. Their use as sanitizer in industrial scale is recent (Beltrame et al. 2012).

Food industries should purpose security limits followed by a monitoring system to assure that the established will be achieved. Some strategies can be cited, mainly the control of concentration of active principles of sanitizing solutions, concentrations of detergents and recommendation of microbiological quality established as technical criterion for sanitized surfaces, processing room, manipulators, and equipments (Andrade et al. 2008).

The efficiency of disinfectant solutions can be measured in terms of *D*-value (Ball 1920; Mazzola et al. 2003). The death rate in presence of constant heat is an exponential function. Consequently, when the log_10_ of survivors numbers are traced as a function of the time, the behavior is described as a line. The *D*-values are defined as the time necessary to the number of viable bacteria to reduce one logarithmic unit. The *D*-values is used as a model for responses aiming at estimating the time necessary for disinfection (10^−3^ CFUmL^−1^) or sterilization (10^−6^ CFUmL^−1^), considering the death kinetic curves as first order (Stumbo 1948a,1948b; Abraham et al. 1990). Furthermore, the *D*-values are suggested as rapid indicators of preservative efficiency of a product (Orth 1979; Akers et al. 1984).

Based on these aspects, the objective of this work was to evaluate the kinetic of inactivation of *L. monocytogenes* in suspension using peracetic acid, chlorhexidine and organic acids as active agent, determining the respective *D*-, *Z*-, and *F*-values. From our knowledge, these important results from an industrial view point are not available in the current literature, mainly for organic acids, pointing out the main contribution of the present work.

## Material and Methods

The lethality kinetic curves for *L. monocytogenes* in different contact times and peracetic acid, chlorhexidine, and organic acids concentrations were obtained and the respective *D*-, *Z*-, and *F*-values were determined.

The strain of *L. monocytogenes* (ATCC 7644), kept in Luria Bertani medium (tryptone 10.0 gL^−1^, yeast extract 5.0 gL^−1^, NaCl 5.0 gL^−1^) at 4°C was subcultured for inoculum preparation in counting standard medium (tryptone 5.0 gL^−1^, yeast extract 2.5 gL^−1^, and dextrose 1.0 gL^−1^) at 35°C for 24 h.

From this inoculum, different dilutions were prepared in peptone distilled water at 0.1% (10^0^ to 10^−8^) and in each replicate of dilution at different concentration of peracetic acid (20% of active principle), chlorhexidine (15% of principle active), or organic acids (blend of ascorbic acid 1.0%, citric acid 0.475%, and lactic acid 0.475%) (0.2, 0.1, and 0.05% v/v, respectively) was added and kept at 25°C. These dilutions were inoculated in counting agar standard (agar 10.0 gL^−1^, tryptone 5.0 gL^−1^, yeast extract 2.5 gL^−1^, and dextrose 1.0 gL^−1^) after different exposure times to disinfectant (0, 0.5, 1, 1.5, 2, 3, 4, 5, 6, 7, 8, 9, 10, 12.5, 15, 18, and 20 min) and incubated at 35°C for 24 h. Dilutions without the addition of the disinfectant were also inoculated in counting agar standard and incubated at 35°C for 24 h to determine the initial number of CFU.

Counting was carried out in the plates from the dilutions with a number of CFU lower than 350 colonies in each exposure to the disinfectant. All determinations were performed in duplicate and the results expressed in terms of mean values.

The mathematical model for determining the *D*-value of *L. monocytogenes* in a fixed concentration of disinfectant was based on differential balance of first order (considering similarities with thermal processes):


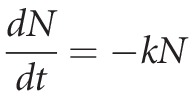
(1)

where *N* is the number of CFU, *t* is the exposure time, and *k* the proportionality constant.

By the integration of equation (1), considering an initial condition of *N* (*t *=* *0) = *N*_0_, where *N*_0_ is the initial number of CFU, we have:


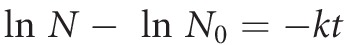
(2)

Rearranging equation (2) in terms of log10 we find:


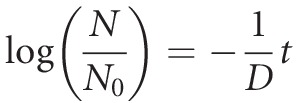
(3)

where *k *=* *1/*D* and *D* is defined as the constant of decimal reduction, which represents the time necessary to reduce a log_10_ cycle along the process.

For obtaining the *D*-value, a linear regression was performed among the different exposure time of the microorganism to the sanitizer and the CFU log_10_ of survivors. Following the procedure, the death resistance constant for *L. monocytogenes* in relation to the disinfectant (*Z*-value) was calculated by equation (4):


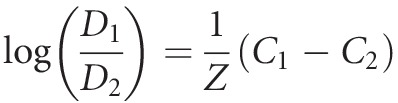
(4)

where *D*_1_ and *D*_2_ are the values of decimal reduction for concentrations *C*_1_ and *C*_2_, respectively. The *Z* constant represents the alteration in concentration necessary to occur a reduction in one log_10_ cycle (90% of reduction) on the death time caused by the disinfectant. For the *Z*-value determination, a linear regression among different chlorhexidine concentration (*C*) and the log_10_ of the respective *D*-value was carried out.

It is worth to mention that equation (3) is extremely important and useful for projects, simulations, and industrial applications of the disinfectant, permitting to obtain the application product concentration. The *F*-value was determined taking into account the *D*-value and the initial and final counting.

## Results and Discussion

The linear regression for the log_10_ of CFU for *L. monocytogenes* and the exposure time to the peracetic acid, chlorhexidine, and organic acids at 0.2, 0.1, and 0.05% permitted to determine the *D*-values, presented in Table 1. Lower *D*-values were obtained for peracetic acid and chlorhexidine, compared with the organic acids (1.18, 1.38, and 5.09 min, respectively).

**Table 1:** Experimental D-values and predictive model for peracetic acid, chlorhexidine, and organic acids at different concentrations.

Sanitizer	Concentration (%)	*D*-value (min)	Predictive model^1^
Peracetic acid	0.20	1.18	*y* = −8.148*X* + 1.658
(*Z*-value = −0.121)	0.10	5.18	*R*^2^ = 0.967
	0.05	21.74	
Chlorexidine	0.20	1.38	*y* = −5.938*X* + 1.266
(*Z*-value = −0.279)	0.10	3.09	*R*^2^ = 0.887
	0.05	12.35	
Organic acids	0.20	6.45	*y* = −1.314*X* + 1.07
(*Z*-value = −3.646)	0.10	8.51	*R*^2^ = 0.994
	0.05	10.23	

1 *y* represents the *D*- value and *X* the correspondent sanitizer concentration.

Mazzola et al. (2003) determined the *D*-values using chlorhexidine for different bacteria. The vegetative strains that showed higher resistance to a solution of chlorhexidine 0.4% were *Enterococcus cloacae* (*D *=* *8.3 min) and *Staphylococcus aureus* (*D *=* *5.9 min) and the most sensible were *Acinetobacter calcoaceticus* (*D *=* *4.1 min), *Serratia marcescens* (*D *=* *4.0 min), and *Escherichia coli* (*D *=* *3.0 min). Exposure times from 3 to 4 min were enough to reduce 90% the population of *E. coli*, *S. marcescens,* and *A. calcoaceticus*. Spores exposed to 2% of chlorhexidine showed *D*-values of 9.1 min for *Bacillus stearothermophilus* and 6.7 min for *Bacillus subtilis*. The same authors verified that the bacteria that presented more resistance to a solution of 1% Minncare (0.45% peracetic acid plus 2.2% of hydrogen peroxide) were *B. stearothermophilus* (*D *=* *9.1 min), *E.coli* (*D *=* *6.7 min), and *B. subtilis* (*D *=* *5.9 min). The most sensitive strains with similar resistance were *A. calcoaceticus* (*D *=* *3.4 min), *E. cloacae* (*D *=* *3.5 min), and *S. aureus* (*D *=* *3.6 min) (Mazzola et al. 2003). Leaper (1984) evaluated the action of peracetic acid under spores of *B. subtilis* and obtained *D*-values of 0.6, 0.9, 3.2, and 25.1 min for concentrations of 0.20, 0.16, 0.12, and 0.08%, respectively.

The linear regression among the log_10_ of *D*-values for *L. monocytogenes* in relation to different peracetic acid, chlorhexidine, and organic acids concentrations showed linearity, giving a *Z*-value of −0.121, −0.279, and −3.646, respectively.

Considering the application of the results presented here to an industrial plant and a counting of 1 × 10^−3^ CFU/cm^2^, for an efficient disinfection (Stumbo 1948a,1948b; Abraham et al. 1990) one can calculate the contact time necessary for achieving the desired result (*F*-value).

Here, for the reduction of 6 log_10_ of *L. monocytogenes* using peracetic acid, at 0.2, 0.1, and 0.05% are necessary 7.08, 31.08, and 130.44 min of contact (*F*-values), respectively. Under the same conditions, we obtained *F*-values of 8.28, 18.52, and 55.87 to chlorhexidine and 30.54, 32.58, and 33.6 to organic acids.

Mazzola et al. (2003) obtained *F*-values from 9 to 12 min for reduction in 3 log_10_ for *E. coli*, *S. marcescens*, and *A. calcoaceticus*, using 0.4% of chlorhexidine.

Beltrame et al. (2012), evaluating different sanitizers, observed that the peracetic acid was efficient at 10°C for all tested microorganisms (*Salmonella choleraesuis, S. aureus, E. coli,* and *L. monocytogenes*), using concentration of 0.2% during 2 min. The chlorhexidine showed efficiency at 0.2% and 2 min for *E. coli*, 18 min for *S. aureus*, and *L. monocytogenes*, but a concentration of 0.5% and 18 min was necessary for *S. choleraesuis*. The organic acids were efficient after 15 min of exposure (0.2% for *L. monocytogenes* and 0.6% for *E. coli* and *S. choleraesuis*), did not showing disinfection under *S. aureus* at the concentration and exposure time evaluated.

**Figure 1:**
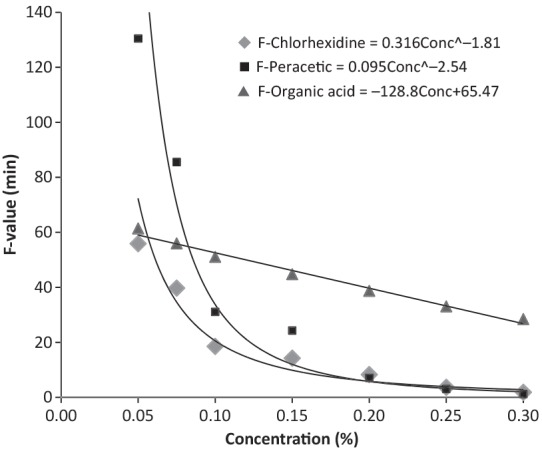
*F*-values and mathematical models obtained for a reduced microbial load of 1.0 × 10^3^ to 1.0 × 10^−3^ CFU/cm^2^ (disinfection) with *D*-values obtained by the predictive models for each different sanitizer.

The analysis of *F*-values was obtained by the mathematical models for each different sanitizer (Fig. 1). we can observe that both chlorhexidine and peracetic acid demonstrated high efficiency in concentrations above 0.15%, compared with the use of organic acids. At concentrations lower than 0.15%, one can verify an exponential increase in *F*-values, for both de chlorhexidine and peracetic acid. The organic acids presented a linear behavior, showing slight variation in *F*-values, is even more effective in under dosage (0.05%). The reduction of peracetic acid and chlorhexidine concentration from 0.2% to 0.05% leads to an increase of 18.4 and 6.7 times on the exposure time necessary to reduce the *L. monocytogenes* concentration from 1.0 × 10^3^ to 1.0 × 10^−3^ CFU/cm^2^. For the organic acids, the same reduction in the concentration results in an increase of only 1.6 times in the exposure time.

Some works in the literature reports a nonlinear death rate for different microorganisms exposed to different disinfectants (Campbell and Dimmick 1966; Turners 1983; Sutton et al. 1991), corroborating the results obtained in the present work.

A predictive model to evaluate the effect of a disinfectant in a nonspecific room is not available and the execution of tests by practical conditions necessary to determine the effect of each product can be difficult. The security of the process as a whole for a specific disinfectant is highly complex and a function of the kind of bacteria, metabolic phase, microorganisms biodiversity, influence of organic material, and processing conditions such as temperature and pH (Asselt and Giffel 2005).

The results obtained here are of fundamental importance in terms of industrial strategy for sanitization procedure, permitting to choose the best relation product concentration/exposure time, aiming at reducing costs without compromising the disinfectant efficiency.

## Conflict of Interest

None declared.
